# Volunteer service duration and career adaptability among Chinese university students: a self-determination theory perspective

**DOI:** 10.3389/fpsyg.2026.1888145

**Published:** 2026-09-30

**Authors:** Cheng Yang, Lidan Tang

**Affiliations:** Department of Psychology, Sichuan Normal University, Chengdu, China

**Keywords:** basic psychological needs satisfaction, career adaptability, motivation, SDT, volunteer service duration

## Abstract

**Introduction:**

Career adaptability has emerged as a crucial resource for university students navigating an increasingly volatile youth employment market. Drawing on Self-Determination Theory (SDT), this study examined the associations among volunteer service duration, basic psychological needs satisfaction, and career adaptability among Chinese university students, as well as the moderating role of initial volunteering motivation.

**Methods:**

A cross-sectional survey was conducted with 581 Chinese university students via an online platform. Participants reported their volunteer service duration and completed the Career Adapt-Abilities Scale–Short Form, the Basic Psychological Needs Satisfaction Scale, and the Simplified Self-Regulation Questionnaire. Data were analyzed using SPSS, the PROCESS macro, and Mplus.

**Results:**

The mediation analysis showed that basic psychological needs satisfaction fully mediated the association between volunteer service duration and career adaptability, such that longer service duration was associated with greater need satisfaction, which, in turn, was associated with greater career adaptability. The subsequent moderated mediation analysis further qualified this pattern. Initial controlled motivation significantly moderated the association between volunteer service duration and basic psychological needs satisfaction, with this association shifting from significantly negative at low controlled motivation to nonsignificant near zero at the mean level and positive but nonsignificant at high controlled motivation. The index of moderated mediation for controlled motivation was significant, whereas the moderating role of autonomous motivation was not.

**Discussion:**

These findings suggest that the association between accumulated volunteering experience and career adaptability may be better understood through the psychological resources associated with volunteering experiences rather than service duration alone, while also indicating that this indirect association may vary according to students’ initial motivational conditions. Universities may therefore benefit from providing sustained volunteering opportunities that support students’ autonomy, competence, and relatedness rather than focusing solely on increasing required service hours.

## 1 Introduction

Amid global economic stagnation and industrial restructuring, youth employment uncertainty has emerged as a formidable challenge (Guo, 2025). Specifically, in China, the rapid expansion of higher education juxtaposes against a contracting labor demand (Xiang et al., 2023), a situation further compounded by AI advancements that curtail traditional entry-level roles (Hui et al., 2024). These pressures lead to salient psychological challenges, most notably career indecision and job-search anxiety (Jin et al., 2024).

Given these increasingly volatile conditions, fostering career adaptability has become a central objective of individual development. Defined as the psychosocial resources and capacities individuals mobilize when confronting career development tasks, transitions, and professional challenges (Ma and Kosnin, 2024), career adaptability is conceptualized within Career Construction Theory not as a static reservoir of capabilities, but as an active process of proactively constructing career paths via psychological regulation (Savickas, 2005). This inherent proactivity renders it a critical competency for university students navigating their academic journeys and transitioning seamlessly into the labor market (Guan et al., 2015). Savickas and Porfeli (2012) operationalized this construct through four interrelated dimensions: concern (future-oriented planning), control (autonomy and responsibility in decision-making), curiosity (exploration of self and environment), and confidence (self-efficacy in overcoming career-related challenges). Extensive empirical evidence indicates that this four-dimensional model is closely linked to effective career exploration and adjustment, serving as a pivotal factor for students navigating career decision-making challenges and transitioning into the workforce (Mou et al., 2026; Alkal, 2025). It is consistently associated with various positive career outcomes, such as higher job satisfaction (Zacher, 2014), better work performance (Savickas, 2005), stronger career commitment (Savickas and Porfeli, 2012), and greater career decision self-efficacy (Hirschi, 2009). Furthermore, cross-cultural research corroborates its robust construct validity and positive developmental implications across diverse contexts (Johnston et al., 2013; Guan et al., 2015; Rudolph et al., 2017). Recent longitudinal evidence further indicates that career adaptability develops dynamically during university and is prospectively associated with students’ academic engagement and achievement, highlighting the importance of examining career adaptability from a developmental perspective (Su et al., 2025).

Career adaptability is not developed solely through traditional classroom education and employment counselling (Carvalho et al., 2023), but also through engagement in rich social practice and experiential learning (Brown et al., 2021). Within the Chinese higher education system, such experiential learning primarily materializes through two distinct pathways: “practical teaching” (e.g., internships) and “social practice” (exemplified by volunteer service; Ministry of Education of the People’s Republic of China, 2012). Internships and practical training are typically defined as highly structured vocational training. While possessing occupational simulation qualities, they are often constrained in practice by stringent performance metrics and fragmented job divisions, driven by enterprises’ instrumental logic of pursuing “economic efficiency maximisation” (Liu et al., 2026; Eraut, 2004). In contrast, volunteer service aligns more closely with informal and non-formal learning frameworks. Characterised by voluntariness, contextual diversity, and rich social interaction, it facilitates career adaptation and skill transfer more effectively than traditional pedagogical approaches (Colley et al., 2003; He et al., 2025). In Western countries, volunteer activities have been shown to be associated with enhanced teamwork, time management, and problem-solving skills (Kanar et al., 2025), while also positively relating to self-efficacy, social responsibility, and career confidence (Astin and Sax, 1998).

Distinct from the spontaneous models prevalent in Western nations, volunteering in China exhibits distinct characteristics of “state mobilisation”. Empirical evidence suggests that participation among Chinese volunteers is often not purely self-initiated but is intricately linked to organisational mobilisation by state institutions or institutional entities, such as universities and government agencies. Consequently, many participants are categorised as “state-affiliated volunteers” (Zhao and Lilly, 2022). In the Chinese context, volunteering is integrated into the higher education system as a form of social practice with salient educational functions. It is commonly managed through credit systems or minimum service hour requirements, which often serve as formal criteria for academic evaluations and graduation (Ministry of Education of the People’s Republic of China, 2015). These institutional arrangements mean that volunteering among Chinese university students is embedded not only in individual behavioral choice but also in an organized educational environment in which participation is formally encouraged, recorded, and recognized (Zhao and Lilly, 2022; Ministry of Education of the People’s Republic of China, 2015). From a social-psychological perspective, such institutional arrangements make volunteering not merely an individual behavioral choice, but a form of participation embedded within a broader social context shaped by organizational structures and social expectations (Zhao and Lilly, 2022). Consequently, compared with contexts in which volunteering is predominantly self-initiated, students embedded in this institutional environment may approach volunteering with a more complex set of considerations, in which personal endorsement and prosocial values coexist with institutional requirements, social expectations, and instrumental concerns. Therefore, although students participate within a similarly structured environment, the extent to which they experience this participation as self-endorsed or externally regulated may differ. This “autonomy within constraints” results in a complex coexistence of motivational orientations, suggesting that student participation is not entirely “purely altruistic” (Wang and Di, 2013).

Nevertheless, volunteering remains a vital avenue for university students to engage in social practice and experience public life. As a form of direct, real-world social practice, volunteering provides a platform for students to immerse themselves in societal dynamics and undergo early “anticipatory adjustment” to professional environments. These experiences enable students to validate their knowledge, accumulate real-world problem-solving experience, and hone organizational skills to enhance overall competence. Volunteering experiences further contribute to shaping positive career values while accumulating vital social capital and intrinsic motivation for future professional development (Hu, 2025; Zhu and Gao, 2025). Consequently, volunteer activities are uniquely associated with career adaptability within the specific context of Chinese higher education.

Despite these documented benefits, an important limitation of the existing literature is that volunteering has often been treated primarily as a participation status or a general experiential context, leaving the role of accumulated exposure insufficiently understood. Whether students participate in volunteering and how long they remain engaged represent theoretically distinct aspects of experience. Participation provides individuals with access to a potentially developmental environment. However, experiential learning theory suggests that competence development does not arise merely from exposure to a single experience; rather, it emerges through a cyclical process in which individuals reflect on concrete experiences, conceptualize what they have learned, and repeatedly test and refine their responses through subsequent practice (Morris, 2020; Tembrevilla et al., 2024). Extending this perspective to volunteering, volunteering is not merely a discrete prosocial act but a process involving antecedents, experiences, and subsequent consequences (Wilson, 2012). Sustained time investment may therefore reflect individuals’ cumulative exposure to real-world tasks, interpersonal collaboration, and organizational norms. Supporting the importance of time investment in volunteering, Hackl et al. (2007) found that the number of hours devoted to volunteering played an important role in explaining its human-capital returns, suggesting that greater time investment may provide more opportunities for skill acquisition and the development of social connections. This temporal dimension may be particularly relevant in the Chinese higher education context, where student volunteering is often embedded within institutional arrangements and volunteer service hours are formally recorded and recognized. Thus, participation time represents not only an individual’s behavioral investment but also a practically meaningful feature of institutionally organized volunteering. Accordingly, the present study focuses on volunteer service duration as a theoretically meaningful behavioral indicator for understanding the development of career adaptability. Examining duration allows us to move beyond the question of whether volunteering is beneficial and instead investigate whether greater cumulative participation is associated with greater career-related adaptive resources. Based on these considerations, we propose:

*H1*: Volunteer service duration is positively associated with career adaptability among university students.

Establishing an association between volunteer service duration and career adaptability, however, would still leave an important question unresolved: what theoretically plausible psychological pathway may help account for this association? Previous studies have documented numerous desirable outcomes of volunteering, including skill development, social connection and self-efficacy (Kanar et al., 2025; Alganami and El Keshky, 2025; Brown et al., 2012). Yet identifying these outcomes does not by itself provide an integrated psychological account of how accumulated volunteering experience may be related to career-related adaptive resources. Greater duration does not necessarily imply greater developmental benefit if the experience itself fails to provide psychologically nourishing opportunities. Therefore, the theoretical significance of volunteer service duration may lie not simply in the accumulation of time, but in the repeated psychological experiences made possible through that accumulated participation.

Self-Determination Theory (SDT) provides a useful framework for understanding this proposed psychological pathway. Compared with traditional behaviorism, which emphasizes external rewards and punishments, and early cognitive theories focusing on information processing, SDT highlights the satisfaction of three basic psychological needs—autonomy, competence, and relatedness—as a fundamental mechanism associated with positive outcomes (Deci and Ryan, 2000). The theory further posits that individuals’ goal content—the value orientation underlying behavior—relates to psychological wellbeing and adaptive development (Deci and Ryan, 2000). Specifically, SDT distinguishes between intrinsic and extrinsic goals. Intrinsic goals are pursued for their inherent value and their capacity to satisfy psychological needs (e.g., personal growth, meaningful relationships, and community contribution), thereby promoting self-integration and psychological fulfilment (Deci and Ryan, 2000; Vansteenkiste et al., 2004). In contrast, extrinsic goals depend on external evaluation, rewards, and social comparison, often eliciting controlled behavior, undermining autonomy, and fostering utilitarian and competitive social orientations; when frustrated, they may also lead to competence frustration and anxiety (Kasser and Ryan, 1996; Niemiec and Ryan, 2009; Sheldon et al., 2001).

Within the Chinese context, university student volunteering typically centers on serving the public good and addressing real-world societal needs, encompassing diverse forms such as supporting the vulnerable and providing social assistance, environmental protection, and support for major public affairs and emergency incidents (Liu, 2010). Such volunteering activities inherently align with an intrinsic goal orientation, constituting highly socially meaningful prosocial activities that embody the quintessential characteristics of intrinsic goals (Stukas et al., 2015; Weinstein and Ryan, 2010). Accordingly, volunteering may constitute a particularly need-supportive experiential context. First, by providing individuals with opportunities for self-expression and the enactment of personal values, individuals achieve “meaning congruence”, a critical source of autonomy satisfaction (Martela and Ryan, 2016). Second, the altruistic nature of volunteering elicits gratitude and positive feedback, which reinforces perceived competence (Martela and Ryan, 2016; Dávila and Díaz-Morales, 2009). Finally, sustained engagement fosters stable interactive relationships with beneficiaries and organizations, thereby relating to the satisfaction of relational needs through meaningful interpersonal connections (Omoto and Snyder, 1995).

Importantly, the theoretical relevance of volunteer service duration may lie in the repeated opportunities it provides for these need-supportive experiences. Longer participation does not merely indicate that students have spent more time volunteering; it also reflects greater cumulative exposure to opportunities for value enactment, mastery, feedback, and interpersonal connection. As these experiences accumulate, they may progressively strengthen students’ satisfaction of autonomy, competence, and relatedness needs. The developmental significance of this need satisfaction extends beyond the volunteering context itself, because these fulfilled needs may constitute transferable psychological resources that support students in coping with future career tasks and uncertainties.

Through this integrated process, the fulfillment of these needs may serve as the vital psychological resources associated with career development (Xu et al., 2023). The satisfaction of autonomy needs signifies that individuals experience freedom of choice and psychological spontaneity in their actions. In this state, individuals typically exhibit higher self-regulation and adaptability (Soenens and Vansteenkiste, 2010), characteristics that relate to an increased capacity to navigate complex professional environments. Secondly, when an individual’s competence needs are satisfied, they are more likely to exhibit confidence, resilience, and proactive exploration when confronting complex occupational tasks. This enhances their performance on the confidence dimension within occupational adaptability (Sönmez et al., 2024). Finally, the fulfilment of relational needs provides ample emotional support and facilitates the establishment of relational networks, helping individuals cultivate a sense of security and belonging. This enables them to respond more flexibly to occupational uncertainties (Song et al., 2022). Basic psychological needs satisfaction may therefore represent a theoretically plausible pathway underlying the association between volunteer service duration and career-adaptive resources. Thus, we propose:

*H2*: Volunteer service duration is indirectly associated with career adaptability through basic psychological needs satisfaction.

Nevertheless, the potentially need-supportive nature of volunteering does not imply that equal amounts of participation will be associated with equivalent psychological benefits for all students. Beyond “what” individuals pursue, SDT also emphasizes “why” individuals engage in a particular activity, that is, the motivational basis underlying their behavior (Deci and Ryan, 2000; Vansteenkiste et al., 2004). Whereas the “what” concerns the content of the activity, the “why” concerns the degree to which engagement is autonomously versus controllingly regulated. Autonomous motivation refers to engaging in an activity with a sense of volition, personal endorsement, and value congruence, whereas controlled motivation refers to engagement driven by external demands, rewards, punishments, social expectations, or internal pressure (Gagné and Deci, 2005; Ryan and Deci, 2017). Accordingly, students may engage in the same volunteering activity—and thus be exposed to the same potentially need-supportive goal content—for fundamentally different motivational reasons.

SDT not only delineates the dual dimensions of goal content (the “what”) and motivational quality (the “why”), but also emphasizes that neither operates in a social vacuum: the “why” of an action reflects the extent to which an individual internalizes the external social environment, playing a pivotal moderating role in how goal content translates into psychological and social adaptation outcomes. For instance, when pursuing wealth as an extrinsic goal stems from highly internalized, socially connected reasons—such as realizing autonomous choices or caring for family—its adverse effects are substantially mitigated compared to when driven by superficial validation (Kasser and Ryan, 1996). Conversely, even when individuals pursue intrinsic goals such as personal growth, the expected psychological benefits are diminished if the enactment is compelled by external social evaluation or interpersonal pressure (Vansteenkiste et al., 2006). This dynamic interplay between goal content and motivational quality indicates that the psychological impact of identical social behavior fundamentally hinges on how individuals construe their relationship with the surrounding social environment. Consequently, although volunteering constitutes a positive, internally oriented activity, the quality of students’ initial motivation may moderate the association between volunteer service duration and basic psychological needs satisfaction.

From the conventional SDT perspective, initial autonomous motivation would be expected to enhance the psychological benefits derived from volunteering. When students enter volunteering with high autonomous motivation, they experience their participation as volitional and self-endorsed, either because the activity is inherently interesting and enjoyable or because its meaning and social value have been sufficiently internalized and integrated with their personal values and sense of self (Ryan and Deci, 2000; Ryan and Deci, 2017). This favorable motivational orientation may enable students to engage more openly and deeply with the need-supportive opportunities afforded by the volunteering context. In this way, autonomous motivation may facilitate the extent to which the social and interpersonal resources embedded in volunteering are associated with the satisfaction of autonomy, competence, and relatedness needs (Gagné and Deci, 2005; Vallerand, 1997; Chirkov et al., 2003). In contrast, students who enter volunteering with controlled motivation—for example, to comply with external demands, fulfill institutional or course requirements, avoid guilt or social disapproval, or obtain favorable evaluations—may experience their participation as more pressuring and less fully self-endorsed (Gagné and Deci, 2005; Ryan and Deci, 2017). Because these external or internal pressures have not been fully internalized and integrated into the self, they may constrain the depth of students’ behavioral engagement and make it more difficult for them to engage with and benefit from the potentially need-supportive resources available in the volunteering environment. Accordingly, controlled motivation may weaken the extent to which participation in a potentially need-supportive social context is associated with basic psychological needs satisfaction.

The present study further considers this relationship from a cumulative-exposure perspective. Autonomous and controlled motivation represent the quality of students’ motivation when they initially engage in volunteering, whereas volunteer service duration reflects the extent of their accumulated participation. This perspective may be particularly informative in the Chinese higher education context, where students’ continued participation unfolds within a volunteering environment that combines institutional requirements and social expectations with opportunities for prosocial contribution, competence development, and interpersonal connection. Thus, even when students initially participate for different reasons, greater service duration represents more extensive involvement in a social context that contains both externally structured and potentially need-supportive elements. Considering volunteer service duration together with initial motivation therefore allows us to examine whether the psychological significance of accumulated participation varies according to why students initially engaged in volunteering. Thus, the question is not only whether different initial motivations are associated with different psychological experiences, but also whether these motivational starting points alter the strength of the relationship between volunteer service duration and basic psychological needs satisfaction.

For students with higher initial autonomous motivation, autonomous prosocial behavior is generally associated with greater basic psychological needs satisfaction and subjective wellbeing (Weinstein and Ryan, 2010). Because these students may experience volunteering as more consistent with their values and self-endorsed reasons from the outset, they may attain relatively high levels of need satisfaction during the early stages of participation. When volunteer service duration is considered, this relatively favorable motivational starting point may leave less room for additional gains in need satisfaction as participation accumulates. Accordingly, the positive association between volunteer service duration and basic psychological needs satisfaction may be relatively weaker among students with higher initial autonomous motivation.

For students with higher initial controlled motivation, a different pattern may emerge. Sheldon and Gunz (2009) introduced the concept of “need hunger” to describe the heightened desire for need satisfaction following need deprivation. When controlled motivation represents the motivational starting point rather than the entirety of students’ subsequent volunteering experience, volunteer service duration may take on particular significance. Students who initially enter volunteering for controlled reasons may nevertheless continue to encounter the intrinsically oriented and potentially need-supportive content of volunteering. This possibility may be particularly relevant in an institutionally structured volunteering context, because the external conditions that initially motivate participation do not necessarily determine the psychological content of the experiences encountered during participation. As their participation accumulates, students may still experience opportunities for meaningful social contribution, value enactment, competence development, and interpersonal connection (Stukas et al., 2015; Weinstein and Ryan, 2010). Their relatively greater “need hunger” may make them more sensitive to these need-supportive experiences as participation accumulates. Consequently, the positive association between volunteer service duration and basic psychological needs satisfaction may be more pronounced among students with higher initial controlled motivation.

Drawing on the interaction between ‘what’ (goal content) and ‘why’ (motivation quality), we propose:

*H3*: Both autonomous and controlled motivations moderate the relationship between volunteer service duration and basic psychological needs satisfaction, albeit with potentially distinct moderating patterns.

Taken together, existing research has provided substantial evidence that volunteering is associated with a range of positive developmental and career-related outcomes, while SDT research has established the importance of basic psychological needs satisfaction and motivational quality for adaptive functioning. However, several questions remain insufficiently understood. In particular, less is known about whether volunteer service duration, rather than volunteering participation more generally, is associated with career adaptability; whether basic psychological needs satisfaction may help account for this association; and whether the strength of these associations varies according to students’ initial motivation. These questions are particularly relevant in Chinese higher education, where volunteering is embedded within a distinctive social and institutional context that may shape both students’ participation and their motivational experiences. Accordingly, the present study examines whether volunteer service duration is indirectly associated with career adaptability through basic psychological needs satisfaction and whether initial autonomous and controlled motivation moderate the association between volunteer service duration and basic psychological needs satisfaction. By integrating these elements, the study seeks to provide a more differentiated understanding of how sustained participation, motivational quality, and basic psychological needs satisfaction are jointly associated with career adaptability within the Chinese higher education context. Based on the theoretical framework outlined above, the present study proposes the following hypotheses:

*H1*: Volunteer service duration is positively associated with career adaptability among university students.

*H2*: Volunteer service duration is indirectly associated with career adaptability through basic psychological needs satisfaction.

*H3*: Both autonomous and controlled motivations moderate the relationship between volunteer service duration and basic psychological needs satisfaction, albeit with potentially distinct moderating patterns.

Based on the above hypotheses, the present study formulated the research theoretical model shown in Figure 1.

**Figure 1:**
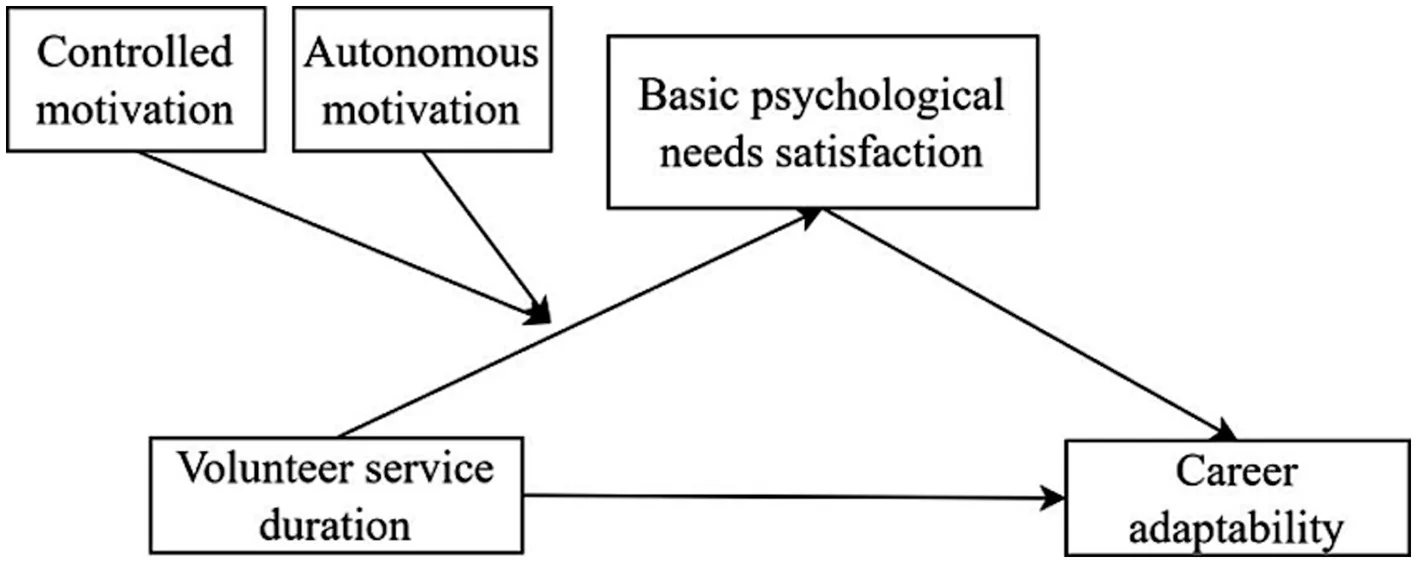
The structural equation model of the moderated mediation (equivalent to Model 7 in Hayes’ PROCESS macro). Hypothesized moderated mediation model linking volunteer service duration to career adaptability through basic psychological needs satisfaction. Autonomous motivation and controlled motivation were proposed as moderators of the relationship between volunteer service duration and basic psychological needs satisfaction.

## 2 Methods

### 2.1 Participants and procedure

An anonymous cross-sectional survey was conducted at a university in China in May 2025. Participants were recruited by distributing the questionnaire link through class group chats. Eligible participants were currently enrolled university students who had participated in voluntary service after entering university. At the beginning of the survey, participants were asked to report their volunteering hours.

Data were collected via Wenjuanxing (Questionnaire Star), a widely used online survey platform for academic research in China. The survey was completely anonymous and voluntary, ensuring that participants’ responses were confidential and that no personally identifying information was collected. To prevent duplicate participation, the questionnaire was bound to participants’ answering devices through Wenjuanxing, allowing each device to submit the questionnaire only once. Therefore, no duplicate responses were identified. All data collection procedures were conducted in accordance with the ethical standards established by the Institutional Review Board of our institution. Electronic informed consent was obtained from all participants before they began the survey. Participants were required to indicate their consent on the online consent page before proceeding to the questionnaire.

A total of 645 responses were initially collected. Cases with one or more missing values (*n* = 7), patterned/inattentive responding (*n* = 28), or zero volunteering hours (*n* = 29) were excluded, resulting in a final sample of 581 participants. Patterned/inattentive responding was defined as exhibiting an obvious response pattern or selecting the same response option for more than five consecutive items. No duplicate submissions were identified.

### 2.2 Research tools

#### 2.2.1 Duration of volunteer activities

In the questionnaire survey, participants were asked to report the cumulative duration of their volunteer activities using a self-reporting method. They were asked, “Up to now, the total duration (in hours) of volunteer activities you have actually participated in since entering college”.

The use of a single-item retrospective measure for volunteer service duration is justified by both the nature of the construct and the Chinese higher education context. Unlike abstract or multidimensional psychological constructs that require multiple indicators to adequately capture their underlying dimensions, volunteer service duration is a concrete, quantitative behavioral metric that can be directly expressed in hours. The operationalization of volunteer involvement in terms of time devoted to volunteering is also consistent with previous research, in which the number of volunteering hours has been used as a quantitative indicator of individuals’ time investment in volunteer activities (e.g., Hackl et al., 2007). Importantly, volunteer service duration is systematically tracked by official platforms in China, such as the China National Volunteer Service System. These formal records serve as a persistent external reference, enabling students to provide accurate retrospective estimates. To further improve the accuracy of the reported duration, participants were instructed in the questionnaire to consult their records on the relevant volunteer-service platform or mini-program and upload a screenshot of their recorded volunteering hours, which was subsequently cross-checked against their reported duration. The screenshot interface displayed only the volunteering-hour record and did not contain names, student IDs, account information, or other personally identifying information; therefore, the verification procedure did not compromise the anonymity of the survey. This procedure provided an additional reference for verifying the reported volunteering hours and supported the reliability of this single-indicator approach.

Among the participants, volunteer service duration had a mean of 33.489 h (SD = 29.953), a median of 21 h, an interquartile range (IQR) of 40 h, and a range of 1–100 h. To examine potential nonlinear effects of volunteer service duration, we conducted a polynomial path analysis by adding a quadratic term (Time^2^) to the structural model. Volunteer service duration was standardized before computing the quadratic term to reduce multicollinearity. Time^2^ was then included as a predictor of both basic psychological needs and career adaptability. The model was estimated using maximum likelihood, with 5,000 bootstrap samples used to obtain 95% bias-corrected confidence intervals. As shown in Table 1, the quadratic effects were nonsignificant, indicating no evidence of significant nonlinear relationships.

**Table 1:** Testing for non-linear effects of volunteer service duration.

Path	Predictor	*B*	SE	*p*	95% confidence interval
Basic psychological needs satisfaction ON	Volunteer service duration (Linear)	−0.542	0.437	0.214	[−1.380, 0.343]
	Volunteer service duration^2^ (Quadratic)	0.435	0.296	0.142	[−0.138, 1.018]
Career adaptability ON	Volunteer service duration (Linear)	0.086	0.419	0.838	[−0.779, 0.872]
	Volunteer service duration^2^ (Quadratic)	0.203	0.294	0.490	[−0.341, 0.816]

#### 2.2.2 Career adaptabilities

The Career Adapt-Abilities Scale-Short Form (CAAS-SF) was used to measure career adaptability (Ma and Kosnin, 2024; Maggiori et al., 2017). The higher the total score of this scale is, the stronger the ability to adapt to career-related changes will be. This scale consists of four dimensions: Concern, Control, Curiosity and Confidence. The CAAS-SF has a total of 12 projects, with 3 questions in each dimension. And it is a Likert 5-point scale (1 = completely inconsistent, 5 = completely consistent). The Cronbach’s alpha for the scale and its four subscales are, respectively, 0.962, 0.910, 0.876, 0.880, and 0.914.

#### 2.2.3 Basic psychological needs satisfaction

Basic psychological needs satisfaction was quantified using the total score of the satisfaction subscale in the Basic Psychological Needs Satisfaction and Frustration Scale (SPNSFS) (Chen et al., 2015). This subscale contains 12 items, which are divided into three dimensions: autonomous needs, relationship needs, and ability needs, with 4 questions in each dimension. The scale is a Likert 5-point scale (1 = completely inconsistent, 5 = completely consistent). The Cronbach’s alpha for the scale and its three subscales are respectively, 0.964, 0.894, 0.906, and 0.922.

#### 2.2.4 Primary motivations for volunteering

This study examines the motivations underlying students’ participation in voluntary service, focusing specifically on their initial motivation levels prior to engagement. Participants were instructed to recall the reasons for which they first began participating in voluntary service after entering university and respond to the following items accordingly. Autonomous motivation was operationally defined as the degree to which students engage in volunteering with a sense of volition and choice, while controlled motivation was defined as engaging due to external or internal pressure. These constructs were measured using the Simplified Self-Regulation Questionnaire (SRQ; Ryan and Connell, 1989). The Chinese version was adapted from Bao (2006) and has demonstrated satisfactory validity across various Chinese research contexts (e.g., Zhou et al., 2023; Liu et al., 2026). Following previous studies that adapted the instructions to specific behavioral contexts (Yang et al., 2018; Zhou et al., 2023), we replaced “studying” in the original instructions with “first engaging in voluntary activities” to fit the context of the present study.

The questionnaire comprises eight items, with four items corresponding to each type of motivation. Controlled motivation is calculated as the total score of the first four items, while autonomous motivation is calculated as the total score of the last four items. All items are rated on a 5-point Likert scale (1 = completely inconsistent, 5 = completely consistent). The Cronbach’s alpha coefficients for controlled motivation and autonomous motivation were 0.822 and 0.934, respectively.

### 2.3 Statistical analysis

A sensitivity power analysis was first conducted using G*Power 3.1 (Faul et al., 2009) to evaluate the adequacy of the final sample size for the most complex regression component of the hypothesized model, namely the incremental effects of the two interaction terms. With a total sample size of 581, an alpha level of 0.05, statistical power of 0.80, two tested predictors, and eight predictors in the full model, the minimum detectable effect size was *f^2^* = 0 0.012. This value is below Cohen’s (1992) conventional benchmark for a small effect (*f^2^* = 0 0.02), indicating that the available sample was sufficiently powered to detect small incremental effects.

Descriptive statistics, independent-samples *t*-tests, one-way analyses of variance (ANOVAs), correlation analyses, and preliminary mediation tests were performed using IBM SPSS Statistics (Version 25.0) and the PROCESS macro (Version 3.3). All analyses conducted in SPSS and PROCESS were based on the original, uncentered variable scores. Subsequently, the proposed moderated mediation model was tested using path analysis in Mplus (Version 8.3). For the Mplus analyses, volunteer service duration, autonomous motivation, and controlled motivation were grand-mean centered prior to model estimation. Interaction terms were then computed using the centered variables. Specifically, the model examined whether autonomous and controlled motivation moderated the first-stage association between volunteer service duration and basic psychological needs satisfaction, and whether the indirect association between volunteer service duration and career adaptability through basic psychological needs satisfaction varied as a function of these two moderators. Model parameters were estimated using the Maximum Likelihood (ML) method. To evaluate the significance of indirect effects, conditional indirect effects, and indices of moderated mediation, 95% bias-corrected bootstrap confidence intervals (CIs) were generated based on 5,000 bootstrap samples.

## 3 Results

### 3.1 Common method Bias test

Since all data in this study were collected via student self-reports, potential common method bias (CMB) was assessed using the unmeasured latent method factor (ULMF) approach, which provides a CFA-based assessment of method-related variance (Podsakoff et al., 2003; Williams et al., 2010). Specifically, we compared the baseline measurement model, in which the 32 items loaded on their respective substantive factors, with a ULMF model in which all 32 items additionally loaded on a common method factor. The method factor was specified to be orthogonal to the substantive factors. As shown in Table 2, adding the method factor yielded negligible improvements in model fit (ΔCFI = 0.034, ΔTLI = 0.032). Both changes were below 0.05, a reference value adopted in previous empirical applications of the latent method factor approach (Liu et al., 2026; Chen, 2007; Bagozzi and Yi, 1990), suggesting that CMB was unlikely to substantially influence the findings.

**Table 2:** Results of the ULMF test.

Model	*X^2^*\df	CFI	TLI	RMSEA	SRMR
Model A (Baseline measurement model)	4.490	0.909	0.902	0.078	0.054
Model B (Model with method facto)	2.256	0.943	0.934	0.046	0.038
Change amount (Δ)	–	0.034	0.032	−0.032	−0.016

### 3.2 Descriptive characteristics of the participants

A total of 581 college students completed the survey and were included in the final analysis. The detailed characteristics of the participants can be found in Table 3.

**Table 3:** Demographic and descriptive characteristics of the participants.

Characteristic	Categories	*N* or mean	% or SD
Gender			
	Female	455	78.313%
	Male	126	21.687%
Grade			
	Freshman	331	56.970%
	Sophomore	83	14.286%
	Junior	160	27.539%
	Senior	7	1.205%
Ethnicity			
	Han Chinese	514	88.468%
	Non-Han ethnic groups	67	11.532%
Residence type			
	Rural	312	53.701%
	Urban	269	46.299%
Type of volunteering activity			
	Educational services	272	46.816%
	Environmental and public welfare	208	35.800%
	Community services	395	67.986%
	Large-scale events or activities	231	39.759%
	Virtual volunteering	57	9.811%
	Healthcare services	49	8.434%
	Other types	67	11.532%
Age		19.750	1.424

Participants typically participated in more than one type of volunteer activity; therefore, this item allowed multiple selections, and the percentages do not sum to 100%.

### 3.3 Demographic differences in the Main study variables

Given the pronounced imbalance in the sample distribution across gender and grade and their potential relevance to the study variables, preliminary analyses were conducted to examine differences in the main study variables across these demographic groups.

As shown in Tables 4 and 5, a significant gender difference was observed only in volunteer service duration, whereas significant grade differences were found across all five main study variables.

**Table 4:** Gender differences.

Variable	Male *M* (SD)	Female *M* (SD)	*F*	*t*
Volunteer service duration	26.095 (27.709)	35.537 (30.256)	6.741	−3.155^**^
Autonomous motivation	15.690 (3.674)	16.110 (3.062)	5.538	−1.300
Controlled motivation	8.087 (3.473)	7.958 (3.361)	1.185	0.397
Basic psychological needs satisfaction	46.413 (9.111)	47.793 (7.770)	8.005	−1.689
Career adaptability	47.540 (8.545)	47.888 (7.813)	1.867	−0.434

*N* = 581. ^*^p < 0.05. ^**^p < 0.01. ^***^p < 0.001. The same convention applies hereafter.

**Table 5:** Grader differences.

Variable	Freshman *M* (SD)	Sophomore *M* (SD)	Junior *M* (SD)	Senior *M* (SD)	*F*	*LSD*
Volunteer service duration	27.207 (25.643)	38.120 (32.708)	43.949 (33.152)	36.571 (37.638)	12.777^***^	④>①, ③>①, ②>①
Autonomous motivation	16.483 (2.820)	15.663 (3.886)	15.344 (3.435)	13.714 (2.215)	6.403^***^	①>④, ①>③, ①>②
Controlled motivation	7.653 (3.247)	8.181 (3.411)	8.531 (3.573)	9.000 (3.651)	2.784^*^	③>①
Basic psychological needs satisfaction	48.187 (7.629)	45.964 (9.308)	47.094 (8.209)	42.000 (7.746)	3.037^*^	①>④, ①>②
Career adaptability	48.456 (7.461)	45.892 (10.270)	47.731 (7.486)	42.000 (6.429)	3.621^*^	①>④, ①>②, ③>④, ③>②

*N* = 581. *p < 0.05. **p < 0.01. ***p < 0.001. The same convention applies hereafter.

### 3.4 Correlation analysis

Pearson correlation analyses were performed to examine the relationships among the primary study variables, including volunteer service duration, autonomous motivation, controlled motivation, basic psychological needs satisfaction, and career adaptability. Gender and grade were also included in the correlation analyses, together with age, which was considered because of its close relationship with grade and its potential to capture additional demographic variation not fully represented by grade alone. The results are presented in Table 6.

**Table 6:** Correlation analysis.

	*M*	SD	1	2	3	4	5	6	7	8
1. Volunteer service duration	33.489	29.953	1							
2. Autonomous Motivation	16.019	3.206	0.142^***^	1						
3. Controlled Motivation	7.986	3.383	−0.075	−0.295^***^	1					
4. Basic psychological needs satisfaction	47.494	8.092	0.089^*^	0.624^***^	−0.295^***^	1				
5. Career adaptability	47.812	7.971	0.098^**^	0.490^***^	−0.232^***^	0.635^***^	1			
6. Age			0.251^***^	−0.091^*^	0.074	−0.074	0.076	1		
7. Gender			0.130^**^	0.054	−0.016	0.070	0.018	−0.021	1	
8. Grade			0.241^***^	−0.174^***^	0.119^**^	−0.087^*^	−0.073	0.649^***^	0.074	1

*N* = 581. *p < 0.05. **p < 0.01. ***p < 0.001. The same convention applies hereafter.

Specifically, volunteer service duration was significantly positively correlated with autonomous motivation (*r* = 0.142, *p* < 0.001), basic psychological needs satisfaction (*r* = 0.089, *p* < 0.05), career adaptability (*r* = 0.098, *p* < 0.01), age (*r* = 0.251, *p* < 0.001), gender (*r* = 0.130, *p* < 0.01) and grade (*r* = 0.241, *p* < 0.001), but was not significantly correlated with controlled motivation (*r* = −0.075, *p* > 0.05).

Autonomous motivation was positively correlated with basic psychological needs satisfaction (*r* = 0.624, *p* < 0.001) and career adaptability (*r* = 0.490, *p* < 0.001), significantly negatively correlated with controlled motivation (*r* = −0.295, *p* < 0.001), age (*r* = −0.091, *p* < 0.05) and grade (*r* = −0.174, *p* < 0.001), but was not significantly correlated with gender.

Controlled motivation was negatively correlated with basic psychological needs satisfaction (*r* = −0.295, *p* < 0.001) and career adaptability (*r* = − 0.232, *p* < 0.001), was positively correlated with grade (*r* = 0.119, *p* < 0.01), but was not significantly correlated with age and gender.

Basic psychological needs satisfaction was significantly positively correlated with career adaptability (*r* = 0.635, *p* < 0.001), but was not significantly correlated with age, gender and grade.

### 3.5 Mediating effect analysis

Mediation analysis was performed using PROCESS Model 4, with age, gender, and grade included as control variables, with grade coded according to year of study (1 = first year, 2 = second year, 3, = third year, and 4 = fourth year) and entered as a single ordinal covariate. The inclusion of these covariates was based on preliminary analyses showing significant gender and grade differences in the study variables, as well as significant correlations between demographic characteristics and several study variables.

The results indicated a significant indirect association between volunteer service duration and career adaptability through basic psychological needs satisfaction. Specifically, the indirect effect was significant, whereas the direct association between volunteer service duration and career adaptability was not significant. As shown in Table 7, the 95% bias-corrected bootstrap confidence interval (CI) for the indirect effect did not include zero.

**Table 7:** The bootstrap 95% confidence interval of the mediating effect path.

Effect	*B*	SE	LLCI	ULCI
Total effect	0.034	0.012	0.012	0.057
Direct effect	0.015	0.009	−0.002	0.033
Indirect effect	0.019	0.007	0.005	0.033

### 3.6 Moderated mediation effects analysis

As shown in Table 8 (after controlling for gender grade and age), the results indicated that controlled motivation significantly moderated the path from volunteer service duration to basic psychological needs satisfaction (*B* = 0.008, *p* < 0.05).

**Table 8:** Path analysis results for the moderated mediation model.

Path	Predictor	*B*	SE	95% confidence interval
Outcome: basic psychological needs satisfaction	Volunteer service duration	−0.003	0.009	[−0.021, 0.016]
	Autonomous motivation	1.526^***^	0.144	[1.237, 1.789]
	Controlled motivation	−0.283^**^	0.100	[−0.495, −0.099]
	Volunteer service duration × Autonomous motivation	0.008	0.005	[−0.001, 0.018]
	Volunteer service duration × Controlled motivation	0.008^*^	0.003	[0.002, 0.015]
	Gender	0.571	0.666	[−0.643, 1.963]
	Grade	0.536	0.406	[−0.255, 1.323]
	Age	−0.301	0.288	[−0.810, 0.292]
Outcome: career adaptability	Basic psychological needs satisfaction	0.526^***^	0.070	[0.384, 0.654]
	Volunteer service duration	0.010	0.009	[−0.007, 0.027]
	Autonomous motivation	0.362^*^	0.173	[0.058, 0.732]
	Controlled motivation	−0.068	0.087	[−0.234, 0.113]
	Gender	−0.693	0.637	[−1.997, 0.516]
	Grade	0.242	0.337	[−0.402, 0.896]
	Age	−0.278	0.222	[−0.728, 0.149]

*N* = 581. *p < 0.05. **p < 0.01. ***p < 0.001. The same convention applies hereafter.

To further interpret this interaction, simple slope analyses were conducted at low (M − 1 SD = −3.38), mean (*M* = 0.00), and high (M + 1 SD = +3.38) levels of controlled motivation (see Figure 2). The association between volunteer service duration and basic psychological needs satisfaction became increasingly positive as controlled motivation increased. Specifically, the simple slope was *b* = −0.030 (95% BC CI [−0.059, −0.004]) at low controlled motivation, *b* = −0.003 (95% BC CI [−0.021, 0.016]) at the mean level, and *b* = 0.024 (95% BC CI [−0.002, 0.057]) at high controlled motivation. Thus, the association shifted from a significant negative association at low controlled motivation to a nonsignificant association at the mean level and a positive but nonsignificant association at high controlled motivation.

**Figure 2:**
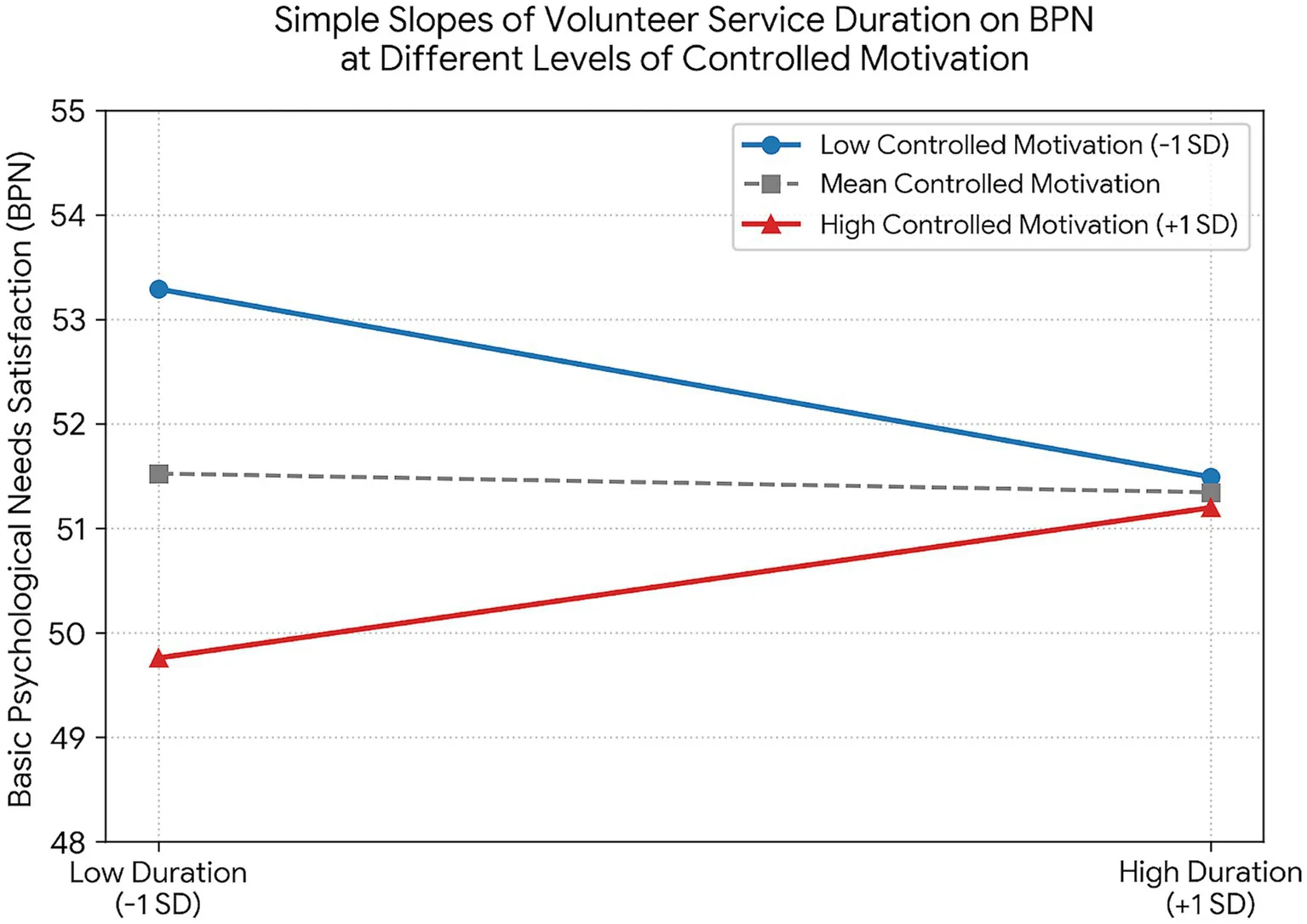
Moderating effect of controlled motivation on the relationship between volunteer service duration and basic psychological needs satisfaction (BPNS). The association was significantly negative at low controlled motivation but positive and nonsignificant at high controlled motivation.

In contrast, the interaction between volunteer service duration and autonomous motivation was not significant (*B* = 0.008, *p* > 0.05).

As shown in Table 9, further analysis indicated that the index of moderated mediation for controlled motivation was significant (95% BC CI [0.001, 0.008]), whereas the index for autonomous motivation was not significant. This result indicates that the indirect association between volunteer service duration and career adaptability through basic psychological needs satisfaction varied significantly as a function of controlled motivation. Specifically, at low controlled motivation, the conditional indirect effect was negative and significant (*B* = −0.016, SE = 0.007, *p* = 0.031, 95% BC CI [−0.032, −0.003]). At the mean level, the conditional indirect effect was nonsignificant (*B* = −0.002, SE = 0.005, *p* = 0.765, 95% BC CI [−0.012, 0.008]). At high controlled motivation, the conditional indirect effect became positive but remained nonsignificant (*B* = 0.013, SE = 0.008, *p* = 0.089, 95% BC CI [−0.001, 0.029]). Overall, the conditional indirect association shifted from significantly negative at low controlled motivation toward a positive direction as controlled motivation increased.

**Table 9:** Index of moderated mediation and conditional indirect effects.

Path	Moderator	Estimate	SE	*p*	95% BC CI
Index of moderated mediation	Autonomous motivation	0.004	0.002	0.086	[0.000, 0.010]
	Controlled motivation	0.004^**^	0.002	0.008	[0.001, 0.008]
Conditional indirect effect	Controlled motivation (Low)	−0.016^*^	0.007	0.031	[−0.032, −0.003]
	Controlled motivation (Mean)	−0.002	0.005	0.765	[−0.012, 0.008]
	Controlled motivation (High)	0.013	0.008	0.089	[−0.001, 0.029]

Conditional indirect effects were evaluated based on 95% bias-corrected bootstrap confidence intervals (CIs); an indirect effect was considered significant when its CI did not include zero. *N* = 581. *p < 0.05. **p < 0.01. ***p < 0.001. The same convention applies hereafter.

## 4 Discussion

This study examined the association between volunteer service duration and career adaptability, the indirect association through basic psychological needs satisfaction, and the moderating role of initial volunteering motivation.

First, H1 was supported, as volunteer service duration was positively associated with career adaptability among university students. However, the magnitude of this association was relatively small, indicating that accumulated volunteer service duration is only weakly related to students’ career adaptability. This finding is broadly consistent with previous research suggesting that sustained participation in volunteering may provide opportunities for skill acquisition, social interaction, and human-capital development (Hackl et al., 2007). At the same time, the modest effect size qualifies a simple “more volunteer service duration is better” interpretation. Volunteer service duration primarily reflects the extent of students’ cumulative exposure to volunteering experiences, but greater exposure does not necessarily guarantee that these experiences will be transformed into career-relevant resources. From an experiential learning perspective, competence development depends not merely on repeated exposure to activities, but on whether individuals reflect on, integrate, and apply what they have experienced across subsequent situations (Morris, 2020; Tembrevilla et al., 2024). Thus, longer service duration may increase opportunities to encounter real-world tasks, interpersonal collaboration, and organizational demands, while the developmental value derived from these opportunities may vary substantially across students. This interpretation also points to the need to examine the psychological processes through which accumulated volunteering experience may become developmentally meaningful.

Second, H2 was supported at the statistical level, as a significant indirect association between volunteer service duration and career adaptability through basic psychological needs satisfaction was observed. Specifically, volunteer service duration was positively associated with basic psychological needs satisfaction, which, in turn, was positively associated with career adaptability, whereas the direct association between volunteer service duration and career adaptability was no longer significant after basic psychological needs satisfaction was taken into account. Although the indirect association was statistically significant, its absolute magnitude was relatively modest, suggesting that volunteer service duration represents only one of multiple factors associated with students’ career development. More importantly, the full mediation pattern suggests that the association between accumulated volunteer service duration and career adaptability may be primarily accounted for by the psychological experiences associated with such participation, rather than by service duration itself. This interpretation is consistent with self-determination theory, which emphasizes satisfaction of the needs for autonomy, relatedness, and competence as a central psychological process through which social environments contribute to adaptive functioning (Deci and Ryan, 2000; Ryan and Deci, 2017). In this sense, the developmental relevance of volunteering may depend less on simply accumulating service hours than on whether continued participation is accompanied by experiences that satisfy students’ basic psychological needs. Importantly, however, this mediation analysis captures the overall indirect association without considering whether it varies across students with different initial motivations, a possibility examined further in the subsequent moderated mediation analysis.

The current institutional frameworks and content design of volunteer services in Chinese universities may provide opportunities for such need-supportive experiences. Compared to highly constrained settings like classrooms or internships, university volunteering retains considerable choice and self-determination despite educational system requirements. Students may select service domains, methods, and participation depth based on interests and strengths (Weinstein and Ryan, 2010; Gagné, 2003; Tang et al., 2019). This sense of autonomy is linked to volition, self-congruence, and psychological integration (Deci and Ryan, 2000; Ryan and Deci, 2017), which are associated with sustained participation, psychological wellbeing (Vecina et al., 2013; Millette and Gagné, 2008), and career adaptation (Douglass and Duffy, 2015; DeFreese and Smith, 2013). Concurrently, volunteering provides rich social connectedness through multi-layered relationships involving teamwork and organizational guidance (Omoto and Snyder, 2002). These positive engagements enhance perceptions of social support and belonging (Martela and Ryan, 2020), bolstering emotional regulation and empathy (Grant, 2008; Post, 2011). Ultimately, these relational resources may help students cultivate a collaborative orientation and resilience, factors positively associated with career adaptability (Xu et al., 2023). Within the Chinese context, social mobilization mechanisms may further reinforce social recognition and group belonging, strengthening the identity associated with voluntary behavior (Zhao and Lilly, 2022). Moreover, during volunteering, individuals may gain immediate satisfaction from task completion; crucially, the relatively non-evaluative nature of this context may reduce fear of failure and strengthen perceptions of competence (Millette and Gagné, 2008). Volunteering can also provide concrete evidence of capabilities through beneficiary feedback and observable environmental or social impact (Weinstein and Ryan, 2010). The satisfaction of competence needs is associated with heightened efficacy, self-confidence, and skills relevant to professional contexts (Xu et al., 2023; Tolentino et al., 2014). Taken together, these autonomy, relatedness, and competence supportive experiences provide a plausible explanation for the overall indirect association between accumulated volunteer service duration and career adaptability through basic psychological needs satisfaction.

The overall indirect-effect pattern observed in the mediation analysis suggests that the developmental relevance of volunteer service duration may not lie in the accumulation of time itself, but in the psychological resources derived from accumulated participation. Volunteer service duration primarily reflects the extent of students’ exposure to volunteering experiences; greater exposure may provide more opportunities for meaningful contribution, interpersonal connection, and competence-building experiences, but time investment itself does not necessarily constitute a developmental resource. Rather, these experiences may become relevant to career adaptability when they are translated into psychological resources such as basic psychological needs satisfaction. This interpretation is consistent with recent evidence showing that longer volunteering participation is associated with specific psychological and social resources, including social connectedness, meaningfulness, personal growth, and confidence (Alganami and El Keshky, 2025; Mbah and Williams, 2025), while research on service-learning further indicates that the perceived quality and utility of the experience are associated with career adaptability (Kanar and Bouckenooghe, 2025). Nevertheless, the full mediation pattern observed in the mediation analysis should not be taken to imply that basic psychological needs satisfaction is the only possible mechanism linking accumulated volunteering experience with career adaptability. Other processes, such as skill acquisition, self-efficacy, career exploration, and social capital, may also contribute to this association and warrant examination in future research.

The modest magnitude of the indirect effect may also reflect the fact that service duration is a relatively coarse indicator of accumulated volunteering experience. Equal amounts of time investment do not necessarily entail equivalent developmental experiences, as students may differ substantially in the quality of their participation, the responsibilities they undertake, the feedback they receive, and the extent to which their volunteering experiences support autonomy, competence, and relatedness. Consequently, variation in service duration alone may capture only a limited portion of the need-supportive experiences that are relevant to career adaptability. Future research could therefore move beyond cumulative service hours by incorporating more differentiated indicators of volunteering experience, such as participation quality, level of responsibility, continuity of engagement, and perceived need support, to clarify which aspects of accumulated participation are most relevant to the development of career adaptability. From a practical standpoint, although the observed indirect effect is modest at the individual level, its potential relevance should be considered in light of the systemic nature of university volunteering in China. In a high-mobilization context involving large numbers of students, even modest associations may have meaningful population-level implications (Funder and Ozer, 2019). However, the observed associations do not imply that simply extending required service hours would improve career adaptability. Universities may benefit more from designing sustained volunteering opportunities that support students’ autonomy, provide meaningful competence feedback, and facilitate genuine interpersonal connection, thereby allowing accumulated participation to be associated with career adaptability through the satisfaction of basic psychological needs. This heterogeneity in volunteering experiences also raises the possibility that the association between accumulated service duration and need satisfaction may differ according to the motivational conditions under which students initially engage in volunteering.

Third, H3 was partially supported. It is important to note that the moderated mediation model further qualified the mediation pattern observed for H2. Whereas the mediation analysis for H2 estimated the overall indirect association without considering moderation, the inclusion of initial autonomous and controlled motivation and their interaction terms in the moderated mediation model allowed this association to vary across levels of initial motivation. Accordingly, the nonsignificant association between volunteer service duration and basic psychological needs satisfaction at the mean levels of the moderators should not be interpreted as contradicting the mediation result for H2. Rather, it indicates that the indirect association identified in the mediation analysis was conditional on students’ initial motivational characteristics. Controlled motivation significantly moderated the association between volunteer service duration and basic psychological needs satisfaction, whereas autonomous motivation did not. This asymmetric pattern differs from a straightforward SDT-based expectation that autonomous motivation would be more likely than controlled motivation to facilitate the psychological benefits associated with sustained participation. Particularly noteworthy is the contrasting role of controlled motivation across the bivariate and interaction results. At the bivariate level, controlled motivation was negatively associated with both basic psychological needs satisfaction and career adaptability, consistent with the general SDT proposition that controlled regulation is associated with less adaptive psychological functioning. Yet, the interaction analysis revealed a more nuanced pattern: as controlled motivation increased, the association between volunteer service duration and basic psychological needs satisfaction shifted in an increasingly positive direction. Specifically, the association was significantly negative at low controlled motivation, nonsignificant and close to zero at the mean level, and positive but nonsignificant at high controlled motivation. Thus, rather than indicating that controlled motivation simply strengthened an already positive association, the findings suggest that controlled motivation altered the direction of the duration–need satisfaction association from negative toward positive. A similar pattern emerged for the conditional indirect association through basic psychological needs satisfaction: the indirect association was significantly negative at low controlled motivation, nonsignificant at the mean level, and became positive at high controlled motivation, where it showed a trend toward significance (*B* = 0.013, *p* = 0.089, 95% BC CI [−0.001, 0.029]). This seemingly paradoxical pattern is theoretically informative. Although controlled motivation was generally associated with lower levels of adaptive psychological functioning, its moderating role revealed a more nuanced picture: as initial controlled motivation increased, the association between accumulated volunteering experience and basic psychological needs satisfaction became progressively more positive, shifting from a negative association at low controlled motivation toward a positive direction at higher levels. This pattern suggests that the role of initial controlled motivation may depend on how subsequent volunteering experiences unfold over time. In particular, because controlled motivation in the present study captured students’ reasons for initially engaging in volunteering, sustained participation may provide opportunities for externally initiated or instrumentally motivated engagement to acquire different psychological meaning through accumulated experience. Accordingly, the observed shift toward a more positive duration–need satisfaction association may be understood in terms of how initially controlled participation interacts with subsequent volunteering experiences.

The significant negative association observed at low initial controlled motivation warrants particular consideration. Importantly, low controlled motivation should not be interpreted as indicating highly autonomous participation, because autonomous and controlled motivation represent distinct dimensions of behavioral regulation (Gagné and Deci, 2005; Ryan and Deci, 2020). Person-centered research has identified a low autonomous–low controlled motivational profile associated with relatively unfavorable outcomes (Van den Broeck et al., 2013). Notably, a similar profile has been identified among Chinese university students and was among the motivational configurations associated with the least favorable outcomes (Ganotice et al., 2022). Although the present analysis did not directly identify such a profile, as autonomous motivation was held at its mean level when controlled motivation was probed, it is possible that some students with low initial controlled motivation also lacked strong autonomous reasons for volunteering. For these students, initial participation may therefore have been supported by neither salient external incentives nor strong personal endorsement, resulting in relatively weak motivational engagement.

The institutionally embedded nature of university volunteering may become particularly relevant as participation accumulates. In the Chinese university context, volunteering is often organized around service-hour expectations, organized activities, and other normative requirements. For students whose initial participation is weakly anchored in either external incentives or autonomous engagement, continued involvement may make these institutional requirements increasingly salient. Although such constraints may not have constituted a strong initial reason for participation, accumulating service obligations may increasingly highlight the externally structured nature of continued participation. Consistent with this possibility, Stukas et al. (1999) found that perceived external control altered the consequences of prior volunteering experience and that mandatory service was particularly unfavorable for students who were initially less inclined to volunteer freely. Similarly, required student volunteers have been found to report weaker internal and stronger external motivation than nonrequired volunteers (Beehr et al., 2010).

From this perspective, the negative association between service duration and basic psychological needs satisfaction at low initial controlled motivation may reflect the increasing salience of institutional constraint among some students with relatively weak initial motivational engagement. As service hours accumulate, continued participation may increasingly be experienced as an obligation to be completed rather than as self-endorsed engagement, potentially undermining the sense of volition central to autonomy need satisfaction and limiting the active engagement through which competence and relatedness are satisfied. Thus, under this motivational condition, longer participation may be associated with lower overall need satisfaction rather than merely an absence of additional psychological benefits. Importantly, the present study did not directly identify a low autonomous–low controlled subgroup or assess changes in perceived institutional constraint or motivation over time. This interpretation therefore remains tentative and requires direct examination in longitudinal research.

On the one hand, this pattern may reflect greater opportunities for psychological gains among students with higher controlled motivation. Individuals with higher controlled motivation often commence participation under external pressure or reward/punishment systems and may be particularly sensitive to structured environmental feedback (Vansteenkiste et al., 2005). For such students, longer volunteer service duration may provide greater exposure to the social interactions and structured feedback embedded in volunteering contexts. Over time, such experiences may provide opportunities to perceive greater task significance (Grant, 2008) and facilitate motivational internalization (Ryan and Deci, 2020; Howard et al., 2021). As demonstrated by the meta-analysis of Vasconcellos et al. (2020), perceived competence emerged as a particularly important correlate of autonomous motivation among the three basic psychological needs. Thus, repeated exposure to environments that provide informational feedback and supportive input could potentially contribute to competence need satisfaction and subsequent motivational internalization.

On the other hand, the stronger duration–need satisfaction association at higher levels of controlled motivation may also reflect an “institutional–psychological” transformation process that is particularly relevant to the Chinese cultural and institutional context. A prominent feature of such a cultural context is the emphasis on the “interdependent self”, where individual behavior is largely regulated by social norms and the expectations of others (Markus and Kitayama, 1991). From the perspective of SDT, this externally referenced mode of behavioral regulation corresponds closely to controlled motivation, which is driven by external contingencies and social expectations (Vansteenkiste et al., 2004). However, within an interdependent cultural framework, social norms themselves often exhibit a high degree of self-relevance and value internalization (Markus and Kitayama, 1991). Consequently, individuals are capable of deeply internalizing “cultural norms” that ostensibly emphasize compliance and external control. When enacting these norms, individuals can still experience a relatively high sense of autonomy, rather than merely feeling coerced (Chirkov et al., 2003). Volunteering represents a specific practice deeply rooted in such social expectations, carrying strong prosocial normative attributes. Furthermore, volunteer practices in China exhibit distinct characteristics of institutional embeddedness, whereby state mobilization and organizational systems provide individuals with structured channels and sustained platforms for participation (Zhao and Lilly, 2022). Under the dual influence of culture and institutional structures, individuals’ deep engagement and integration of volunteering behaviors may foster the satisfaction of basic psychological needs. Consistent with this perspective, a longitudinal study by Zhang and Huang (2025) further indicated that controlled motivation is associated with a normative identity processing style and is linked to egocentric helping behaviors (which conceptually aligns with the current study’s focus on engaging in volunteer activities for instrumental reasons, such as academic credits). This suggests that, within such settings, controlled motivation can still manifest as actual helping behaviors. These findings further imply that, even when initially driven by controlled motivation, individuals may still become embedded in social contexts through sustained participation. Such sustained engagement may provide a context in which basic psychological need satisfaction and motivational internalization are more likely to occur, although these processes were not directly examined in the present study.

Taken together, these explanations suggest that the moderating role of controlled motivation does not necessarily contradict its negative overall association with adaptive functioning. Rather, students who initially participate for more controlled reasons may have greater opportunities for psychological gains through sustained volunteering experiences, such that the association between accumulated service duration and need satisfaction shifts progressively toward a more positive direction as initial controlled motivation increases. In particular, sustained participation may provide greater opportunities for initially externally regulated engagement to acquire personal meaning and become internalized, especially within an institutional context that provides structured and continued opportunities for participation. Importantly, however, the positive simple slope at high controlled motivation did not reach statistical significance; therefore, the present findings should be interpreted as evidence of a significant change in the duration–need satisfaction association across levels of initial controlled motivation, rather than as evidence of a significant positive association at high controlled motivation.

Finally, a potential boundary condition of this pattern concerns the characteristics of the volunteering environment. From an SDT perspective, the shift toward a more positive association between volunteer service duration and need satisfaction as initial controlled motivation increases may depend on whether sustained participation occurs in a context that provides autonomy support, meaningful social interaction, and constructive feedback. In less supportive or more controlling volunteering environments, longer participation may not show the same increasingly positive association with need satisfaction and could even be associated with less favorable psychological experiences. Because the present study did not directly assess these contextual characteristics, this possibility remains a theoretical proposition rather than an empirically tested boundary condition. Future research could directly examine whether the quality and need-supportive features of volunteering environments condition the interaction between service duration and initial motivation.

In contrast, the moderating effect of autonomous motivation did not reach statistical significance. Importantly, this null interaction should not be interpreted as evidence that autonomous motivation is irrelevant to volunteer service duration or that students with higher autonomous motivation have limited potential to benefit from longer participation. In the present study, autonomous motivation was strongly and positively associated with basic psychological needs satisfaction, suggesting that its role may be more evident in its overall association with need satisfaction than in altering the strength of the duration–need satisfaction association. From an SDT perspective, autonomous motivation reflects engagement based on personally endorsed values, interests, or a sense of volition (Ryan and Deci, 2020), and such motivation has generally been associated with more adaptive psychological experiences (Sheldon et al., 2001; Vansteenkiste et al., 2004). One possible interpretation is therefore that students with higher autonomous motivation may experience volunteering as relatively self-congruent and need-supportive across different levels of participation, leaving less variation in the association between accumulated volunteer service duration and need satisfaction. However, the present data do not directly test this explanation, nor do they provide evidence of a ceiling effect or diminishing marginal returns among highly autonomously motivated students. Future longitudinal research examining changes in motivation and need satisfaction across different levels and durations of volunteering is needed to evaluate these possibilities.

Taken together, the present study adds to the volunteering and career development literature by considering volunteer service duration, basic psychological needs satisfaction, and initial motivation within an integrated model. Rather than treating longer volunteer service duration as inherently beneficial, the findings suggest that its association with career adaptability may be better understood by considering the satisfaction of basic psychological needs. The different roles observed for controlled and autonomous motivation further indicate that students’ initial reasons for volunteering may be relevant to how accumulated volunteer service duration is associated with basic psychological needs satisfaction. In particular, the significant moderating role of controlled motivation suggests that the duration–need satisfaction association may vary in both magnitude and direction depending on students’ initial controlled motivation. From an SDT perspective, these findings provide a more differentiated understanding of the relationships among volunteer service duration, motivational quality, need satisfaction, and career adaptability, particularly in the institutionally embedded context of Chinese university volunteering. Practically, the findings suggest that universities may benefit from attending not only to students’ service hours but also to the need-supportive quality of volunteering experiences, so that students with different initial motivations have opportunities to derive meaningful psychological resources from participation. These implications should be regarded as considerations for the design of future interventions rather than as evidence supporting mandatory volunteering requirements.

Some demographic differences were also observed in the present study. Gender differences were limited to volunteer service duration, with female students reporting longer cumulative volunteer service duration than male students. Gender differences in volunteering are not uncommon among Chinese college students; for example, Geng et al. (2022) found that volunteering participation varied by gender, suggesting that male and female students may differ in their engagement with volunteering. However, the present study did not examine the factors underlying this difference in accumulated volunteering time, and it should therefore be interpreted cautiously. Grade differences were more pervasive, with significant differences observed across all five study variables. Students in higher grades generally reported longer volunteer service duration, which is unsurprising because volunteer service duration was measured as the total number of service hours accumulated since university entry, giving students in later years a longer period in which to accumulate volunteering experience. The differences in motivation across grades may further indicate that students’ reasons for volunteering vary across stages of university study. Consistent with this possibility, Martin et al. (2025) found that lower-level undergraduate students reported stronger career-development motivation than upper-level students. Thus, the observed grade differences may reflect not only unequal opportunities to accumulate service hours but also differences in students’ reasons for participation across stages of university study. Nevertheless, these demographic differences were not the primary focus of the present study, and their underlying mechanisms cannot be determined from the current data.

## 5 Limitations and future directions

This study has several limitations. First, the cross-sectional design precludes causal inference and leaves open the possibility of reverse relationships and unmeasured confounding. The relatively modest effect sizes also suggest that the present model captures only part of the factors associated with career adaptability. Future research should employ multi-wave longitudinal designs and incorporate a broader range of individual and contextual factors to clarify the temporal relationships among these variables.

Second, cumulative volunteer service duration provides only a coarse measure of volunteering involvement. Because duration was assessed as total service hours since university entry, students in higher grades had greater opportunities to accumulate service hours. Although grade and age were statistically controlled, such adjustment cannot fully account for differences in exposure opportunity. Moreover, the present survey did not collect precise information on time enrolled (e.g., months since university entry), precluding a more explicit exposure-adjusted analysis. Future research should collect precise information on time enrolled and distinguish cumulative service duration from participation intensity per unit of time. In addition, although activity type was assessed, students could select multiple categories and hours were not recorded separately for each activity type. Thus, the present data cannot reliably determine whether different types of volunteering are differently associated with career adaptability. Nor did the study assess activity–career alignment, participation regularity or intensity, or the quality of volunteering experiences. Future research should therefore incorporate activity-specific service hours, career relevance, participation patterns, and experience quality to provide a more differentiated assessment of volunteering involvement.

Third, autonomous and controlled motivation, basic psychological needs satisfaction, and career adaptability were assessed using self-report measures from the same participants. In particular, initial autonomous and controlled motivation were assessed retrospectively by asking participants to recall their reasons for initially engaging in volunteering after entering university. Such retrospective reports may be susceptible to recall and reconstructive biases, as subsequent volunteering experiences may influence how participants remember or interpret their initial motivations. Although our assessment of common method variance did not indicate substantial method effects under the specified model, this procedure cannot eliminate the limitations associated with single-source measurement. In addition, career adaptability was assessed as a self-perceived psychosocial resource rather than through objective career-related behavioral indicators. Future research could integrate multiple data sources, including evaluations from volunteer supervisors or instructors and objective or behavioral indicators of career development.

Finally, the sample was characterized by substantial gender and grade imbalance and was drawn from a single university in China, which constrains the generalizability of the findings. Female students constituted 78.31% of the sample, meaning that the estimated associations may more strongly reflect the volunteering experiences and psychological processes of female students and should not be assumed to generalize equally to male students. This concern is particularly relevant given the observed gender differences in some of the focal variables. The grade distribution was also markedly uneven, with first-year students comprising 56.97% of the sample and only seven fourth-year students being represented. Consequently, the overall estimates may disproportionately reflect students at earlier stages of university study, while the experiences of students approaching graduation are insufficiently represented. This is especially relevant because significant grade differences were observed in the focal variables and because opportunities to accumulate volunteer service hours inherently increase with time enrolled. Although gender, grade, and age were statistically controlled in the main analyses, statistical adjustment can reduce potential confounding but cannot make an imbalanced sample demographically representative or establish that the proposed relationships operate equivalently across demographic groups. Future studies should therefore recruit more gender- and grade-balanced samples across multiple universities and examine whether the proposed mediation and moderation processes are replicated across gender and different stages of university study.

## 6 Conclusion

This study employed a moderated mediation framework to examine the associations among volunteer service duration, basic psychological needs satisfaction, initial motivation, and career adaptability among Chinese university students. Volunteer service duration was positively but modestly associated with career adaptability, suggesting that accumulated participation was related to students’ career adaptability, although the magnitude of this association was limited. Basic psychological needs satisfaction fully mediated this association: greater volunteer service duration was associated with higher need satisfaction, which, in turn, was associated with greater career adaptability, whereas the direct association between service duration and career adaptability was no longer significant after need satisfaction was taken into account. The subsequent moderated mediation analysis further qualified this mediation pattern by showing that the indirect association was not uniform across motivational conditions. Specifically, initial controlled motivation, rather than autonomous motivation, significantly moderated the association between volunteer service duration and basic psychological needs satisfaction. As initial controlled motivation increased, the duration–need satisfaction association shifted from significantly negative at low controlled motivation, to nonsignificant and close to zero at the mean level, and then to positive but nonsignificant at high controlled motivation. Correspondingly, the indirect association between volunteer service duration and career adaptability through basic psychological needs satisfaction also varied significantly as a function of initial controlled motivation. These findings suggest that although basic psychological needs satisfaction accounted for the overall association between volunteer service duration and career adaptability in the mediation analysis, this indirect association was further conditioned by students’ initial controlled motivation.

These findings extend the application of self-determination theory to institutionally embedded volunteering by distinguishing accumulated participation from the psychological experiences associated with that participation and by showing that the relevance of these experiences may differ according to students’ initial reasons for volunteering. In particular, the different roles of controlled and autonomous motivation highlight the importance of considering not only how much students participate, but also the motivational conditions under which their participation begins and develops. Practically, universities should not assume that simply increasing required service hours will necessarily be associated with greater career adaptability. Greater attention may instead be given to creating sustained volunteering experiences that provide meaningful opportunities for autonomy, competence, and interpersonal connection, including for students who initially participate for externally regulated reasons. Nevertheless, given the cross-sectional, single-university design and the specific institutional and cultural context of Chinese higher education, the findings should not be interpreted causally, and their generalizability to other student populations, volunteering systems, and cultural contexts requires further examination.

## Data Availability

The raw data supporting the conclusions of this article will be made available by the authors, without undue reservation.
